# Specific Inhibition of CYP4A Alleviates Myocardial Oxidative Stress and Apoptosis Induced by Advanced Glycation End-Products

**DOI:** 10.3389/fphar.2019.00876

**Published:** 2019-08-09

**Authors:** Rui Wang, Li Wang, Jinlong He, Shanshan Li, Xiaojing Yang, Pengyuan Sun, Yuhui Yuan, Jinyong Peng, Jinsong Yan, Jianling Du, Hua Li

**Affiliations:** ^1^College of Pharmacy, Dalian Medical University, Dalian, China; ^2^Department of Hematology, Liaoning Medical Center for Hematopoietic Stem Cell Transplantation, Liaoning Key Laboratory of Hematopoietic Stem Cell Transplantation and Translational Medicine, the Second Hospital of Dalian Medical University, Dalian, China; ^3^Collaborative Innovation Center of Tianjin for Medical Epigenetics and Department of Physiology and Pathophysiology, Tianjin Medical University, Tianjin, China; ^4^Institute of Cancer Stem Cell, Dalian Medical University, Dalian, China; ^5^Department of Endocrinology, the First Affiliated Hospital of Dalian Medical University, Dalian, China

**Keywords:** advanced glycation end-products, CYP4A, myocardium, oxidative stress, apoptosis

## Abstract

High exposure to advanced glycation end-products (AGEs) may induce cardiotoxicity. However, the effects and mechanisms remain to be further clarified. CYP4A plays an important role in the pathophysiological process of myocardial abnormalities by modulating oxidative stress and apoptosis (OS/Apop) signaling pathway. The present work aimed to investigate whether CYP4A mediates AGEs-induced myocardial injury. AGEs solution was administered intragastrically to C57BL/6 mice for 60 days, while the specific inhibitor of CYP4A, HET0016, was given from the 47th day *via* intraperitoneal injection for 2 weeks. Levels of OS/Apop in heart tissue were measured. The effects on the cell viability and apoptosis were detected in primary rat cardiomyocytes. To further investigate the mechanism, H9c2 cells were treated with HET0016 or small interfering RNAs (siRNAs) against CYP4a mRNA before incubation with AGEs. Exposure to AGEs led to significantly increased expression of CYP4A and levels of OS/Apop in heart and H9c2 cells both *in vivo* and *in vitro*. The OS/Apop pathway was activated with increased expression of NOX2, p-JNK, and cleaved caspase-3 (c-caspase-3) and decreased expression of p-Akt and Bcl-xL both *in vivo* and *in vitro*. Specific CYP4A suppression by HET0016 or siRNA exerted significant protective effects by attenuating AGEs-induced OS/Apop pathways *in vitro*. Our results demonstrate that specific inhibition of CYP4A might be a potential therapeutic option for myocardial injury induced by AGEs.

## Introduction

Advanced glycation end-products (AGEs) are heterogeneous compounds formed by nonenzymatic glycation and the oxidation of proteins and lipids *via* Maillard reactions (Delgado-Andrade et al., 2007). Endogenous AGEs are generated gradually during aging. The process is substantially accelerated during diabetes (Huebschmann et al., 2006; Vlassara and Uribarri, 2014). Exogenous AGEs are derived to a great extent from the modern diet, prepared or processed under high temperatures, stored for long periods, or containing various additives (Scheijen et al., 2016; Snelson and Coughlan, 2019). AGEs play important roles in the development of diabetes and its complications, including nephropathy and micro- and macro-vascular diseases. Recent studies showed that AGEs also promote the development of diabetic cardiomyopathy. AGEs may induce oxidative stress, inflammation, endoplasmic reticulum stress, autophagy, and extracellular matrix reactions, leading to myocardial injury, systolic and diastolic dysfunction, and even heart failure (Rajesh et al., 2012; Bodiga et al., 2014; Wang et al., 2017b; Jia et al., 2018; Pei et al., 2018). Nevertheless, most *in vivo* studies are performed in diabetic subjects, and there are many other factors, such as impaired cardiac insulin signaling, cardiac metabolic abnormalities, and endogenous AGEs that are involved in the progression of diabetic cardiomyopathy (Zhang et al., 2018). These factors and their interactions with AGEs need to be considered when the roles of AGEs are interpreted in these investigations. Recently, several studies indicated that long-term intake of excessive dietary AGEs leads to myocardial injury and cardiac dysfunction prior to the occurrence of diabetes, strongly suggesting that AGEs alone may induce cardiotoxicity (Cai et al., 2014; Clarke et al., 2016; Deluyker et al., 2016). Nevertheless, the effects and mechanisms of AGEs on the myocardium remain far from clear and require further research efforts.

CYP4A, an ω-CYP hydroxylase in the CYP450 family, plays an important role in many pathophysiological processes, including cancer metastasis, renal fibrosis, hepatic steatosis and fibrosis, and diabetic complications (Park et al., 2014; Chen et al., 2017; Wang et al., 2017a; Zhang et al., 2017; Zhou et al., 2018). CYP4A is highly expressed in myocardium and vasculature and is a primary contributor to the pathogenesis of cardiovascular diseases (CVD) (Park et al., 2014). CYP4A mediates endothelial dysfunction and hypertension (Singh et al., 2007; Inoue et al., 2009). Inhibition of CYP4A resulted in profound reduction in myocardial infarction size in ischemia-reperfusion injuries in canine hearts (Nithipatikom et al., 2004). Other investigations showed that inhibition of CYP4A significantly reduced cardiomyocytes apoptosis in myocardial ischemia-reperfusion by inhibiting reactive oxygen species (ROS) production and the ERK1/2 signaling pathway (Lv et al., 2008; Han et al., 2013). CYP4A also mediated isoprenaline-stimulated cardiomyocyte apoptosis *via* the mitochondrial-dependent pathway (Jiang et al., 2017). These studies suggest that CYP4A may induce myocardial injury by modulating OS/Apop signaling pathway.

The present study aimed to investigate the effects of AGEs on myocardium by long-term intragastric administration of AGEs solution and to determine whether CYP4A mediates AGEs-induced myocardial effects.

## Materials and Methods

### Preparation of AGEs

AGEs were prepared by the nonenzymatic glycation reaction system for D-glucose according to a published protocol. The reaction mixtures contained 50 g/L bovine serum albumin (BSA) (Sigma, St. Louis, MO, USA), 0.5 mol/L D-glucose, 0.5 mmol/L ethylenediaminetetraaceticacid (EDTA) and 1.5 mmol/L of phenylmethanesulfonyl fluoride (PMSF) in 0.2 M PBS, pH 7.2. The mixtures were filtered, sealed, incubated in biochemical incubator kept at 37°C and dark for 90 days. Unincorporated sugars were removed by dialysis against a large volume of PBS. The endotoxin concentrations of all mixtures were tested by Endpoint Chromogenic LAL Assays (Yeasen, Shanghai, China), which were less than 0.5 EU/ml, which were safe for mouse gavage and cell culture. Control BSA was incubated in the absence of reducing sugars and under identical conditions. AGEs content was determined spectrofluorometrically (370 nm excitation and 440 nm emission) and was expressed as the percentage of relative fluorescence compared with control BSA (Hao et al., 2011; Kim et al., 2015).

### Animal Experiments

Adult male C57BL/6 mice (6 to 8 weeks) weighing from 16 to 20 g were obtained from the Animal Center of Dalian Medical University (Dalian, China). All procedures were performed according to the Institutional Animal Care and Use Committee guidelines and approved by the Institutional Ethics Committee. The mice were kept in a specific pathogen-free grade animal facility with a 12-h light-dark cycle. Thirty-six adult male C57BL/6 mice were randomly divided into four groups (*n* = 9): 1) BSA control group (Con), 2) Con+HET0016 (Cayman Chemical, USA) group, 3) AGEs group, and 4) AGEs+HET0016 group. All C57BL/6 mice underwent intragastric administration of AGEs (500 mg/kg/day) or the same dosage of BSA as a control for 60 consecutive days. At 14 days before the end of the experiments, mice received intraperitoneal injections of the specific CYP4A inhibitor, HET0016 (2.5 mg/kg/d) in the Con+HET0016 and AGEs+HET0016 groups or vehicle in the BSA control and AGEs groups (Jiang et al., 2004; Thomas et al., 2005; Cai et al., 2014; Guo et al., 2015; Wang et al., 2019).

### Rat Cardiomyocytes Isolation and Culture

Cardiomyocytes were isolated and cultured from ventricles of neonatal rats (1–2 days old) as described previously (Louch et al., 2011; Guo et al., 2018). Briefly, heart ventricles were minced immediately, washed with cold PBS, and digested with trypsin and collagenase type II. Fibroblasts were depleted twice by pre-plating the cells for 90 min at 37°C in maintenance medium. The cardiomyocytes were then grown and cultured in 10% fetal bovine serum (FBS)/dulbecco’s modified eagle medium (DMEM) medium.

### H9c2 Cell Culture

H9c2, a rat cardiomyocyte line, was obtained from the Institute of Biochemistry Cell Biology (Shanghai, China). H9c2 cells were cultured in high-glucose DMEM supplemented with 10% FBS, 100 U/ml of penicillin and 100 mg/ml streptomycin at 37°C in a humidified atmosphere of 5% CO_2_.

### Cell Treatments

In the experiments with the specific inhibitor of CYP4A, H9c2 cells were divided into four groups: 1) BSA control group (Con), 2) BSA+HET0016 group (Con+HET0016), 3) AGEs group, and 4) AGEs+HET0016 group. H9c2 cells at a density of 1 × 10^5^ cells per well were seeded on six-well plates and treated at the time of 90% confluence. HET0016 or vehicle in FBS-free media (2 μM) was added 1 h before exposure to 10 μM AGEs or BSA for 24 h (Adamopoulos et al., 2016; Zhang et al., 2018; Wang et al., 2019).

In the experiments with siRNAs inhibition, H9c2 cells were divided into four groups: 1) BSA+siControl (siCon), 2) BSA+siRNAs (siRNAs), 3) AGEs+siControl (AGEs+siCon), and 4) AGEs+siRNAs. H9c2 cells at a density of 1 × 10^5^ cells per well were seeded on six-well plates and transfected at 70–80% confluence with siRNAs against rat CYP4a1, CYP4a2, CYP4a3 (**Table 1**), or nonbinding control siRNA. Transfection was performed with Lipofectamine 2000 (Invitrogen, Karlsruhe, Germany) according to the manufacturer’s instructions. After 24 h of CYP4a siRNAs transfection, cells were treated with 10 μM AGEs or BSA for 24 h (Park et al., 2014).

**Table 1:** Synthetic primers for siRNAs.

Gene name	Sense sequences/antisense (5′-3′)
CYP4a1	F: GCA GGU CAA GAC UCC UCU ATT R:UAG AGG AGU CUU GAC CUG CTT
CYP4a2	F: GCG GAC UCU GUC AGU AUA ATT R:UUA UAC UGA CAG AGU CCG CTT
CYP4a3	F: CCU GUU GAA UGG GAA GAA ATT R: UUU CUU CCC AUU CAA CAG GTT
Negative control	F: UUC UCC GAA CGU GUC ACG UTT R: ACG UGA CAC GUU CGG AGA ATT

### RNA Extraction and Real-Time qPCR Analysis

The total RNA was extracted using TRIzol reagent following the manufacturer’s instructions (Takara, Dalian, China), and the purity of the RNA samples was analyzed. Then, total RNA was used to prepare cDNA by reverse transcription using the cDNA Synthesis SuperMix kit (TransScriptTM, Dalian, China). The obtained cDNA was amplified using synthetic primers (**Table 2**) specific for CYP4a1, CYP4a2, CYP4a3, and β-actin, as described in the instructions for the TransStart Top Green qPCR SuperMix kit (TransScriptTM, Dalian, China). The RT-PCR reactions were performed in the following conditions: 1 cycle of denaturation at 94°C for 30 s, 40 cycles of denaturation at 94°C for 5 s, and annealing at 60°C for 34 s. The data (fold-changes in the Ct values for each of the genes) were analyzed using the 2^−ΔΔCt^ method.

**Table 2:** Synthetic primers for real-time PCR.

Gene name	Forward sequence/Reverse sequence (5′-3′)
CYP4a1	F: TTGTCAACTTGCCCATGATCA R: CTGTCCCCATTCTCCATTCTG
CYP4a2	F:CTCGCCATAGCCATGCTTATC R:CCTTCAGCTCATTCATGGCAATT
CYP4a3	F:CAGAGTCTTGGGACAATGGACA R:GGCATACATTAATTTCACCATGAGA
β-actin	F: CCAGATCATGTTTGAGACCTTCAA R: GTGGTACGACCAGAGGCATACA

### Western-Blot Analysis

Heart tissues, free of blood, were homogenized and lysed with ultrasonic cell disruptor. H9c2 cells and heart homogenates were lysed with ice-cold RIPA (radio immunoprecipitation assay) lysis buffer containing phenylmethanesulfonyl fluoride (PMSF) (1 mmol/L) for 30 min. Then the lysates were centrifuged at 12,000 rpm for 15 min at 4°C, and the supernatant was collected. The concentrations of protein were determined by a Pierce BCA (bicincjoninic acid) protein assay kit. Equal amounts of proteins (25 μg for H9c2 cells protein and 40 μg for heart tissues protein) were separated with 10–15% sodium dodecyl sulfate polyacrylamide gel electrophoresis (SDS-PAGE), then transferred to a polyvinylidene fluoride (PVDF) membrane (Millipore, Bedford, MA, USA), and blocked with 5% milk. The specific primary antibodies were against CYP4A (1:300, sc-271983, SANTA CRUZE, USA), p-Akt (1:1,000, #4060, Cell Signaling Technology, USA), Akt (1:1,000, #4691, Cell Signaling Technology), p-JNK (1:500, #9251, Cell Signaling Technology, USA), JNK (1:1,000, #9252, Cell Signaling Technology, USA), NOX2 (1:1,000, ab80508, Abcam, UK), cleaved caspase-3 (1:1,000, AF1150, Beyotime, China), Bcl-xL (1:1,000, AB126, Beyotime, China), and β-actin (1:2,500, 60008-1-Ig, Proteintech Group, China). After washing, the membranes were incubated with the appropriate secondary antibodies. The membranes were then exposed to enhanced chemiluminescence-plus reagents (Amersham Biosciences, Little Chalfont, UK). Emitted light was recorded using a multispectral imaging system (UVP, California, USA), and gels were analyzed using a Gel-Pro Analyzer, Version 4.0 (Media Cybernetics, Rockville, USA).

### Flow Cytometry Analysis of Intracellular ROS

Intracellular ROS were detected using the 2,7-dichlorodihydrofluoresceindiacetate (DCFH-DA) ROS probe (Beyotime Institute of Biotechnology, China). Briefly, H9c2 cells were incubated with the probe (10 μM) for 30 min at 37°C in the dark. Measurements of the fluorescence were performed by FACS Calibur Flow Cytometer (BD Biosciences, San Jose, CA).

### Myocardial LDH, SOD Activity and MDA, AGEs Content Assay

Blood samples were obtained from the abdominal cavity and centrifuged at 3,000 rpm for 15 min, and then serum was collected. The lactate dehydrogenase (LDH) activity and malondialdehyde (MDA) content were determined using an assay kit (Nanjing Jiancheng Corp, China), according to the manufacturer’s recommendations. The level of superoxide diamutase (SOD) activity in the myocardial tissues was quantified by SOD assay kit (Beyotime Institute of Biotechnology, China) according to the manufacturer’s protocol. Serum AGEs were measured using a fluorescence microplate reader (EnSpire, Corning, USA) at an excitation wavelength of 370 nm and an emission wavelength of 440 nm, with BSA as the standard. The quantity of AGEs was expressed in fluorescent units (FUs), which were calculated according to a standard curve of freshly prepared BSA FU values. The fluorescent value of 1 µmol/L BSA was set as 1 FU.

### Cell Viability Assay

Cell viability in cardiomyocytes and H9c2 cells was detected using cell counting kit 8 (CCK8) following manufacturer’s protocol. Cells were seeded at a density of 1 × 10^4^ cells per well in a 96-well culture plate, and three dosages of AGEs (2 μM, 5 μM, and 10 μM) was added in. After culturing for 24 h, the cells were subsequently incubated with CCK8 solution (Bimake, USA) at 37°C for 2 h. The absorbance was measured at 450 nm with a microplate spectrophotometer.

### TUNEL Staining

Terminal-deoxynucleoitidyl transferase mediated nick end labeling (TUNEL) staining of cardiomyocytes, H9c2 cells, and mouse heart tissue sections was performed with the In Situ cell death detection kit (Roche, Germany) according to manufacturer’s instruction. TUNEL-positive cells were photographed under fluorescent microscope (Olympus IX81, Japan) and detected with Imaging Analysis Software (ImageJ, USA).

### Statistical Analysis

All values are presented as the means ± S.E.M. At least three independent experiments (ran in duplicates) were performed for all *in vitro* studies. Statistical analyses were performed with SPSS software (version 20.0; SPSS, Inc, Chicago, IL), and graphs were generated using GraphPad Prism 5 (La Jolla, CA). Differences were considered statistically significant at a *p* value. All data passed normality of distribution. For >2-group comparisons, if data passed normality and equal variance tests, one-way ANOVA with a Tukey *post hoc* test at *p* < 0.05 was used. Otherwise, if data passed normality but could not pass equal variance tests, Dunnett T3 test was performed, and statistical significance was accepted at *p* < 0.05. Statistical results are specified in the figure legends.

## Results

### Inhibition of CYP4A With HET0016 Reduced Cardiac Oxidative Stress Induced by AGEs in Mice

**Figure 1:**
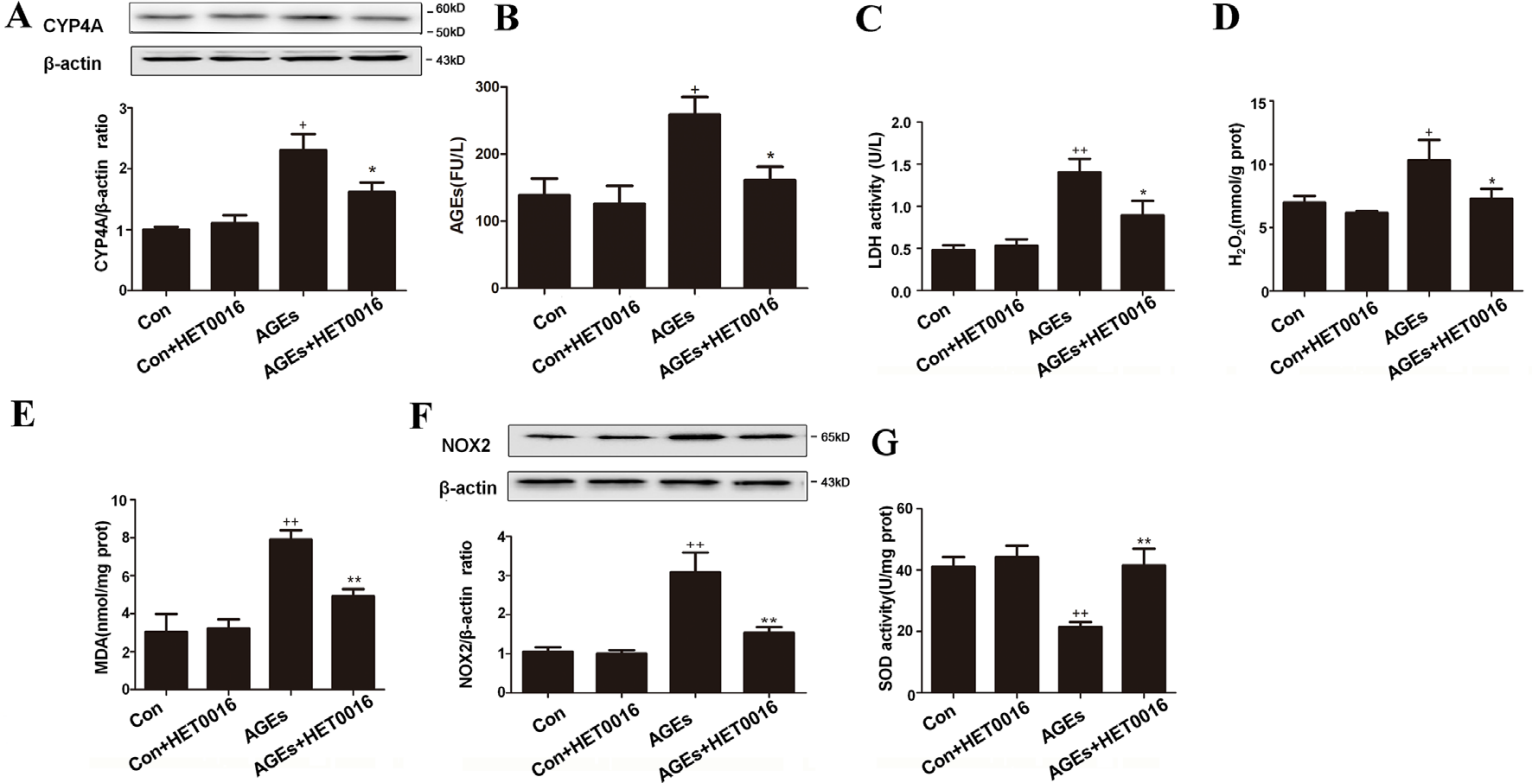
Inhibition of CYP4A with HET0016 reduces myocardial oxidative stress induced by advanced glycation end-products (AGEs) in mice. **(A)** Protein expression of CYP4A, *n* = 3. **(B)** Serum AGEs levels, *n* = 9. **(C)** Serum lactate dehydrogenase (LDH) levels, *n* = 9. **(D)** H_2_O_2_ content in the myocardium, *n* = 9. **(E)** Myocardial malondialdehyde (MDA) content, *n* = 9. **(F)** Protein expression of NOX2, *n* = 3. **(G)** Activity of SOD in the myocardium, *n* = 9. AGEs solution was administered intragastrically to C57BL/6 mice for 60 days, while the specific inhibitor of CYP4A, HET0016, was given from the 47th day *via* intraperitoneal injection. Con, control. Compared with the Con group, *+p* < 0.05, *++p* < 0.01; compared with the AGEs group, *p < 0.05, **p < 0.01 (one-way ANOVA with Tukey *post hoc*).

To investigate whether AGEs is involved in the development of cardiac dysfunction, we detected the cardiac function by ultrasonic cardiogram (**Supplementary Table 1**). Diastolic left ventricle posterior wall thickness (LVPW) was increased in AGEs group compared to control group, slightly moderated in AGEs+HET group. Fractional shortening (%FS) was significantly reduced in the AGEs group and up-regulated in the AGEs+HET group. These data indicated that AGEs treatment induced mild cardiac dysfunction. We harvested heart tissues and blood from mice. As shown in **Figure 1**, CYP4A expression was significantly higher in the AGEs group than in the control group (**Figure 1A**). In AGEs group mice, serum AGEs levels and LDH activity, cardiac H_2_O_2_ and MDA content, and NOX2 expression were higher compared to the control group, and the cardiac SOD activity was lower compared to the control group. HET0016, the specific inhibitor of CYP4A, significantly reversed these AGEs-induced changes (**Figures 1B–G**).

### Inhibition of CYP4A With HET0016 Reduced Cardiac Apoptosis Induced by AGEs in Mice and Neonatal Rat Cardiomyocytes

**Figure 2:**
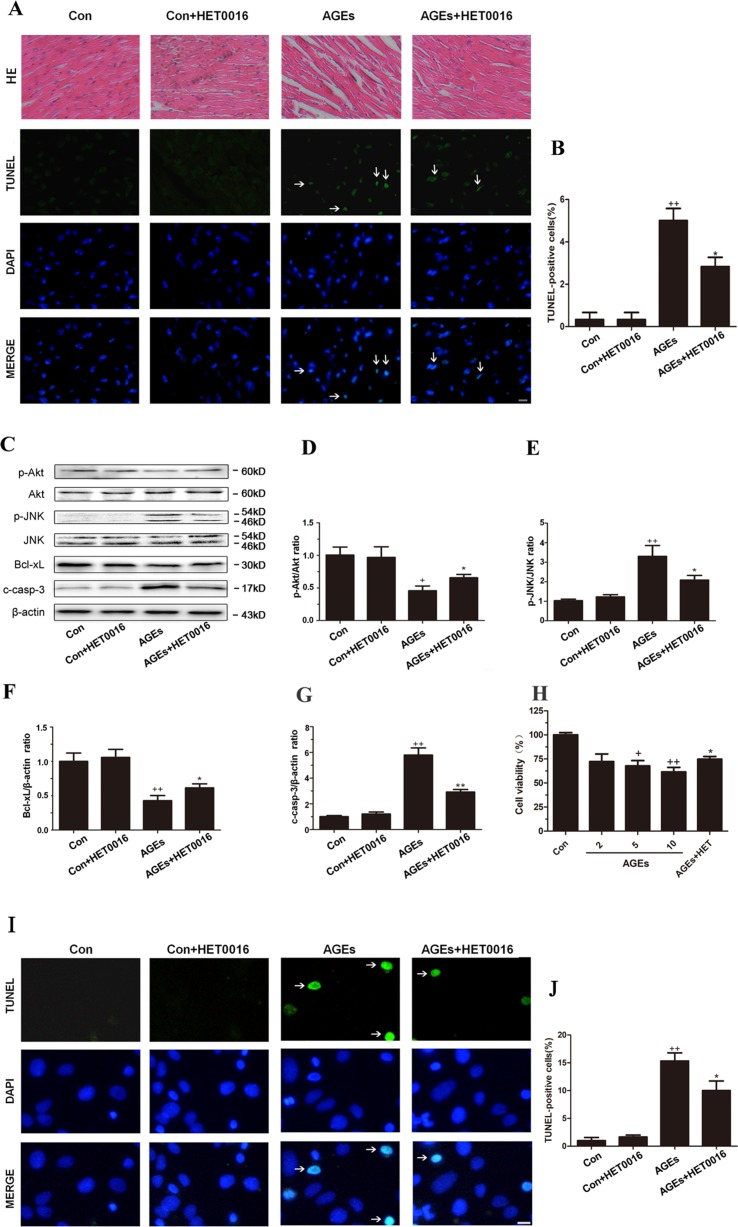
Inhibition of CYP4A with HET0016 reduces myocardial apoptosis induced by AGEs in mice and cardiomyocytes. **(A)** Hematoxylin and eosin (HE) staining and TUNEL staining of mouse heart tissues. Scale bar = 50 μm. **(B)** Relative apoptosis rates are represented as TUNEL-positive cells/DAPI-positive cells, *n* = 6. **(C–G)** Protein expression of p-Akt, p-JNK, Bcl-xL, and c-caspase-3. *n* = 3. **(H)** Cell viability of rat cardiomyocytes detected with cell counting kit 8. *n* = 6. **(I)** Apoptosis of rat cardiomyocytes *via* TUNEL staining. **(J)** Relative apoptosis rates are represented as TUNEL-positive cells/DAPI-positive cells, *n* = 3. Cardiomyocytes were isolated and cultured from ventricles of neonatal rats. Scale bar = 10 μm. AGEs solution was administered intragastrically to C57BL/6 mice for 60 days, while the specific inhibitor of CYP4A, HET0016, was given from the 47th day *via* intraperitoneal injection. Con, control. Compared with the Con group, *+p* < 0.05, *++p* < 0.01, *+++p* < 0.001; compared with the AGEs group, *p < 0.05, **p < 0.01 (one-way ANOVA with Tukey *post hoc*).

Apoptosis has been increasingly proved to be closely associated to oxidative stress, which is a key factor contributing to myocardial injury. Therefore, we examined apoptosis markers in the hearts of AGEs-induced mice. The number of TUNEL-positive cells in mice heart tissue was higher after AGEs treatment. Phosphorylation levels of Akt were markedly lower, and its downstream signaling activity, reflected by the expression of anti-apoptotic factor Bcl-xL, was suppressed. p-JNK and c-caspase-3 were substantially enhanced. AGEs-induced changes were effectively inhibited by HET0016 treatment in mice (**Figures 2A–G**). The cell viability was lower, and the number of TUNEL-positive cells was higher in AGEs-treated cardiomyocytes. HET0016 treatment effectively inhibited above mentioned changes (**Figures 2H–J**).

### Inhibition of CYP4A With HET0016 Protected H9c2 Cells From AGEs-Induced Oxidative Stress

**Figure 3:**
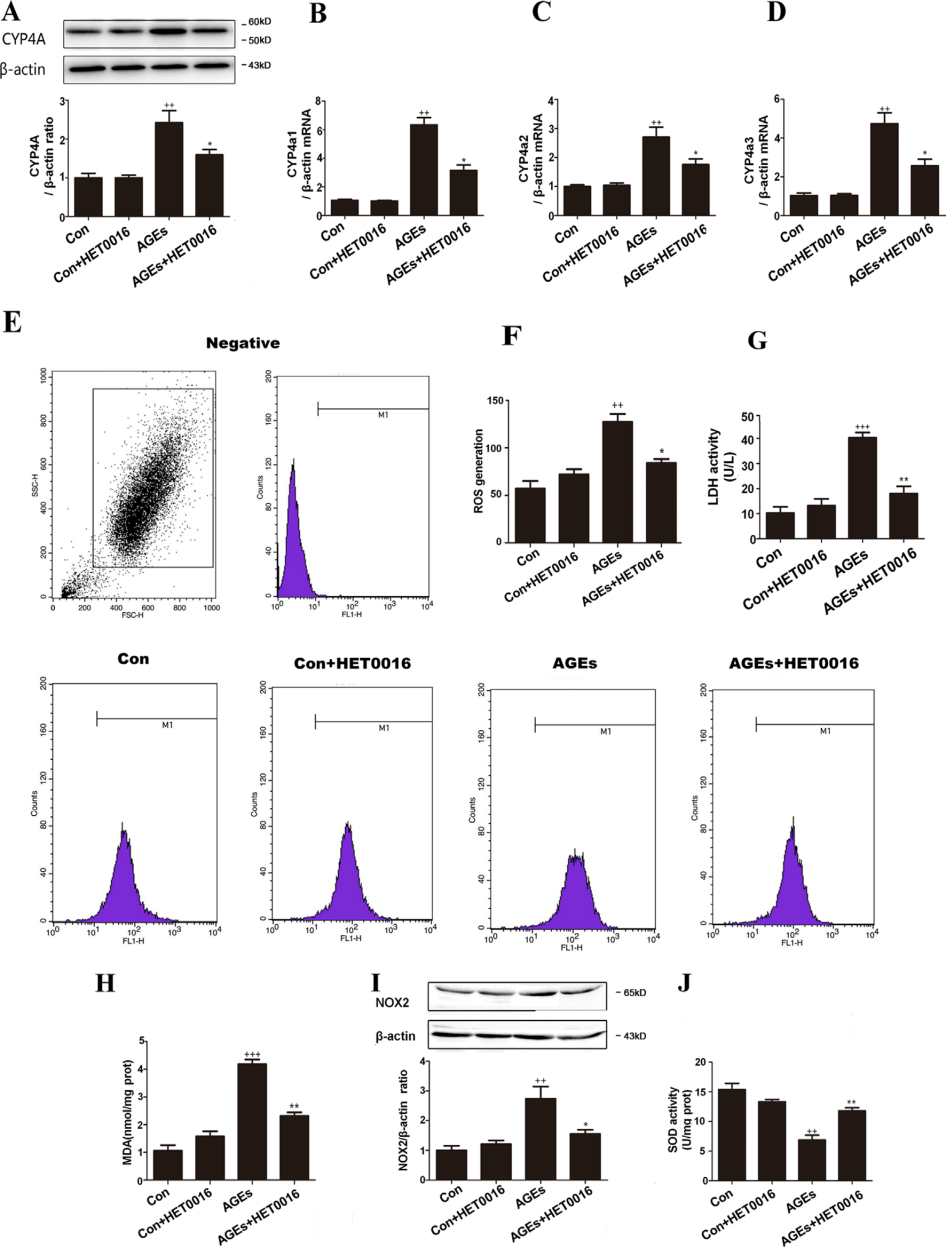
Inhibition of CYP4A with HET0016 protects H9c2 cells from AGEs-induced oxidative stress. **(A)** Protein expression of CYP4A, *n* = 3. **(B–D)** mRNA expressions of CYP4a1, CYP4a2, CYP4a3, *n* = 3. **(E)** Level of intracellular ROS generation. **(F)** A summary of flow cytometry analyses of cells stained with 2,7-dichlorodihydrofluoresceindiacetate (DCFH-DA), *n* = 3. **(G)** LDH activity in the medium, *n* = 6. **(H)** Content of MDA, *n* = 6. **(I)** Protein expression of NOX2, *n* = 3. **(J)** Activity of SOD, *n* = 6. H9c2 cells at a density of 1 × 10^5^ cells per well were seeded on six-well plates and treated at the time of 90% confluence. HET0016 or vehicle in fetal bovine serum (PBS)-free media (2 μM) was added 1 h before exposure to 10 μM AGEs or bovine serum albumin (BSA) for 24 h. Con, control. Compared with the Con group, *++p* < 0.01, *+++p* < 0.001; compared with the AGEs group, *p < 0.05, **p < 0.01 (one-way ANOVA with Tukey *post hoc*).

To further investigate the underlying mechanism, we examined OS markers in H9c2 cells. Protein expression of CYP4A and genes expression of CYP4a1, CYP4a2, and CYP4a3 in H9c2 cells were remarkably higher in the AGEs group but were lower with HET0016 treatment (**Figures 3A–D**). LDH activity, ROS generations, MDA content, and NOX2 expression were significantly up-regulated, and SOD activity was down-regulated in the AGEs group compared to the control group. HET0016 treatment significantly reversed these AGEs-induced changes (**Figures 3E–J**).

### Inhibition of CYP4A With HET0016 Protected H9c2 Cells From AGEs-Induced Apoptosis

**Figure 4:**
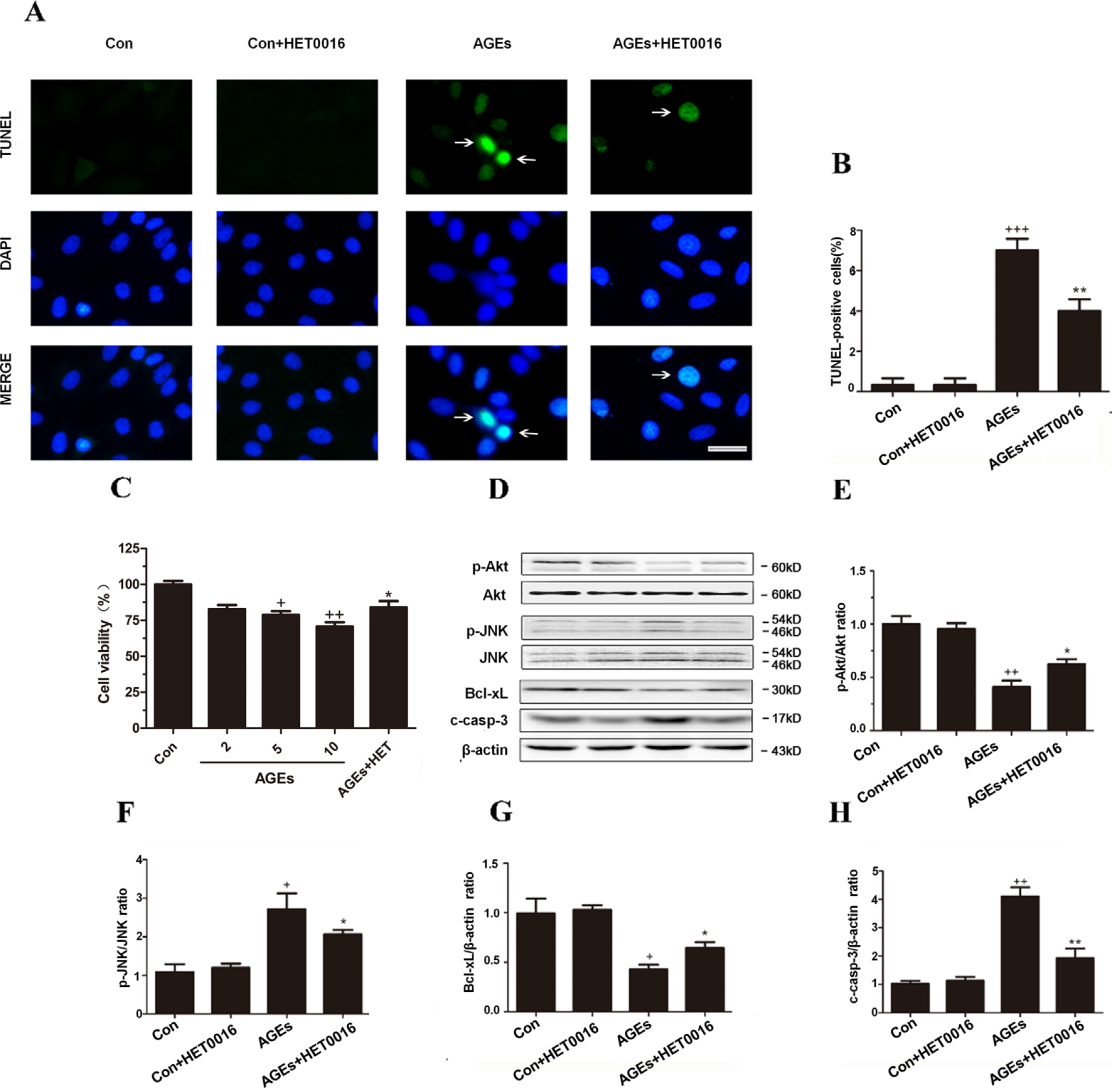
Inhibition of CYP4A with HET0016 protects H9c2 cells from AGEs-induced apoptosis. **(A)** Apoptosis *via* TUNEL staining. Scale bar = 10 μm. **(B)** Relative apoptosis rates are represented as TUNEL-positive cells/DAPI-positive cells, *n* = 3. **(C)** Cell viability detected with cell counting kit 8. *n* = 10. **(D**–**H)** Protein expression of p-Akt, p-JNK, Bcl-xL, and c-caspase-3, *n* = 3. H9c2 cells at a density of 1 × 10^5^ cells per well were seeded on six-well plates and treated at the time of 90% confluence. HET0016 or vehicle in FBS-free media (2 μM) was added 1 h before exposure to 10 μM AGEs or BSA for 24 h. Con, control. Compared with the Con group, *+p* < 0.05, *++p* < 0.01, *+++p* < 0.001; compared with the AGEs group, *p < 0.05, **p < 0.01 (one-way ANOVA with Tukey *post hoc*).

Additionally, we used TUNEL staining to observe the apoptosis on H9c2 cells. The number of TUNEL-positive cells was higher and the cell viability was lower in AGEs-treated H9c2 cells, and HET0016 treatment effectively inhibited above mentioned changes (**Figures 4A–C**). Furthermore, the expression of phosphorylated-Akt and Bcl-xL was significantly reduced in the AGEs group accompanied by enhanced p-JNK and c-caspase-3 expression. HET0016 pretreatment inhibited these AGEs-induced changes (**Figures 4D–H**).

### Inhibition of CYP4A by Small Interfering RNA Protected H9c2 Cells From AGEs-Induced Oxidative Stress

**Figure 5:**
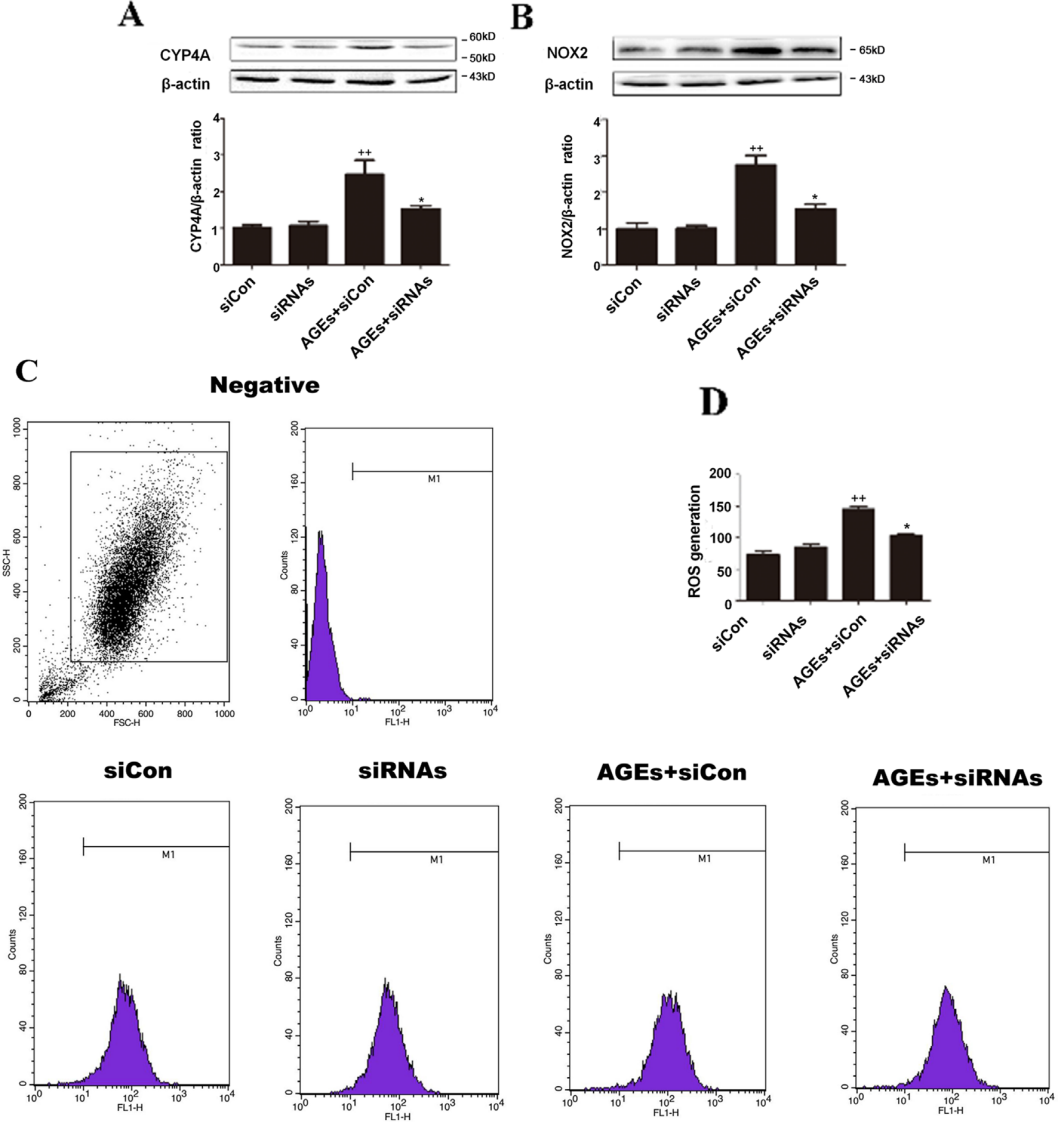
Inhibition of CYP4A by siRNAs protects H9c2 cells from AGEs-induced oxidative stress. **(A)** Protein expression of CYP4A. **(B)** Protein expression of NOX2. **(C)** Level of ROS generation. **(D)** A summary of flow cytometry analyses of cells stained with DCFH-DA. *n* = 3. siCon: siControl. siRNAs: siRNAs against CYP4a1, CYP4a2, and CYP4a3. H9c2 cells at a density of 1 × 10^5^ cells per well were seeded on six-well plates and transfected at 70–80% confluence with siRNAs against rat CYP4a1, CYP4a2, CYP4a3, or nonbinding control siRNA. Transfection was performed with Lipofectamine 2000. After 24 h of Cyp4a siRNAs transfection, cells were treated with 10 μM AGEs or BSA for 24 h. Compared with the siCon group, *++p* < 0.01; compared with the AGEs+siCon group, *p < 0.05, **p < 0.01 (one-way ANOVA with Tukey *post hoc*).

To further elucidate the roles played by inhibition of CYP4A, we induced loss of CYP4A function by small interfering RNA (siRNA). In rat cardiomyocytes, there are three CYP4A isoforms: CYP4a1, CYP4a2, and CYP4a3; as shown in **Figure 5**, a combined treatment of rat CYP4a1 siRNA, CYP4a2 siRNA, and CYP4a3 siRNA effectively reduced AGEs-induced increases in CYP4A and NOX2 and the generation of ROS in rat H9c2 cells, whereas the nonspecific control siRNA treatment had no effects (**Figures 5A–D**).

### Inhibition of CYP4A by Small Interfering RNA Protected H9c2 Cells From AGEs-Induced Apoptosis

**Figure 6:**
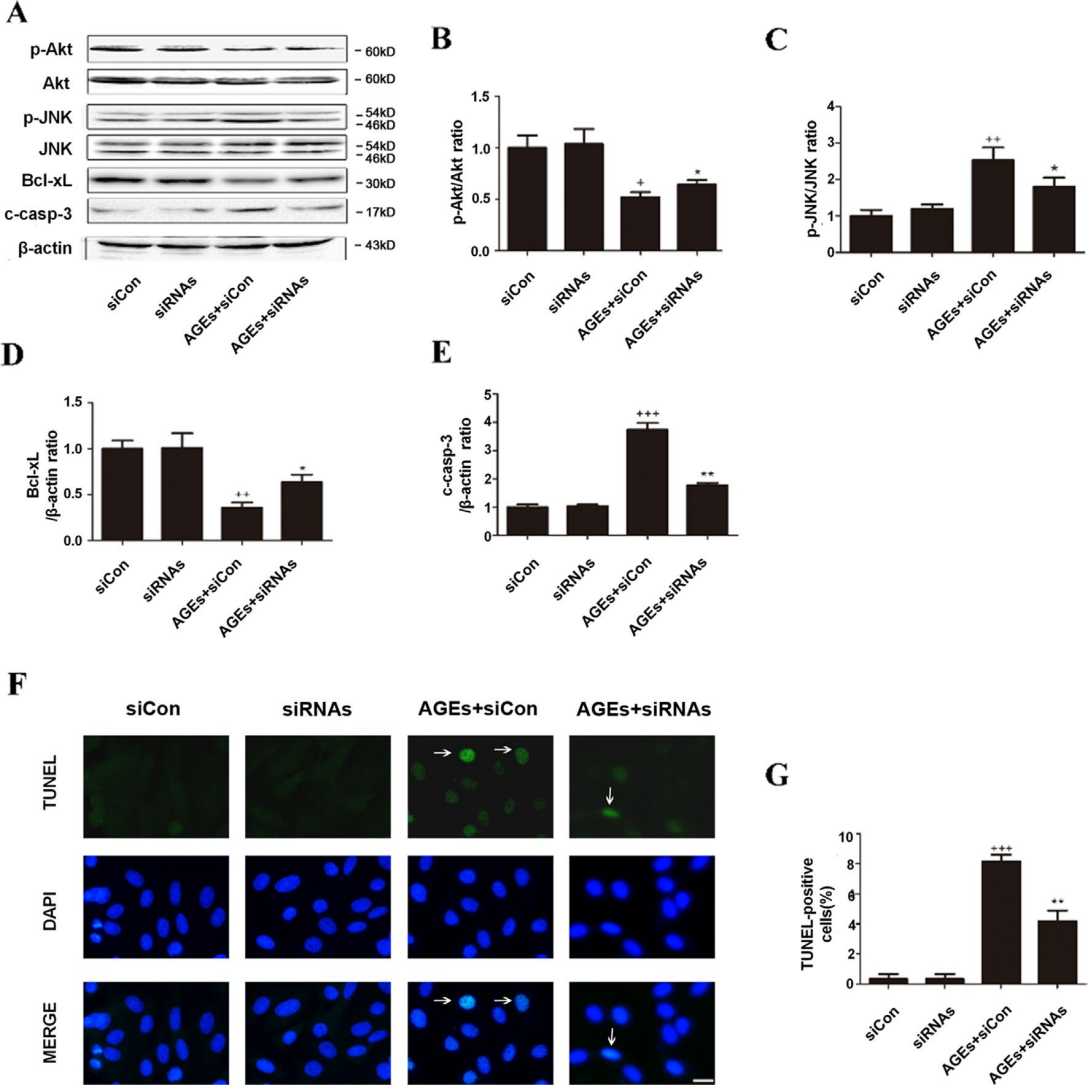
Inhibition of CYP4A by siRNAs protects H9c2 cells from AGEs-induced apoptosis. **(A**–**E)** Protein expression of p-Akt, p-JNK, Bcl-xL, and c-caspase-3, *n* = 3. **(F)** Relative apoptosis rates are represented as TUNEL-positive cells/DAPI-positive cells, *n* = 6. **(G)** Apoptosis *via* TUNEL staining. H9c2 cells at a density of 1 × 10^5^ cells per well were seeded on six-well plates and transfected at 70–80% confluence with siRNAs against rat CYP4a1, CYP4a2, CYP4a3, or nonbinding control siRNA. Transfection was performed with Lipofectamine 2000. After 24 h of CYP4a siRNAs transfection, cells were treated with 10 μM AGEs or BSA for 24 h. Scale bar = 10 μm. siCon: siControl. siRNAs: siRNAs against CYP4a1, CYP4a2, and CYP4a3. Compared with the siCon group, *^+^* *p* < 0.05, *^++^* *p* < 0.01, *^+++^* *p* < 0.001; compared with the AGEs+siCon group, *p < 0.05, **p < 0.01 (one-way ANOVA with Tukey *post hoc*).

As shown in **Figure 6**, all changes in the apoptosis pathway induced by AGEs, including the downregulated phosphorylated Akt and Bcl-xL, the upregulated p-JNK, c-caspase-3, and TUNEL-positive cells, were all blocked significantly with siRNAs against CYP4a1, CYP4a2, and CYP4a3, whereas the nonspecific siRNA control had no significant effects (**Figures 6A–G**).

These results suggest that CYP4A specifically mediate AGEs-induced myocardial OS/Apop, and inhibition of CYP4A is important to alleviate myocardial OS/Apop.

## Discussion

AGEs take part in the development of many chronic diseases. Previous studies primarily focused on the pathogenic role of endogenous AGEs in diabetes and its complications. Nevertheless, many other co-existing factors and their interactions with AGEs in patients with diabetes may exert unexpected influences on the outcomes of these studies. Since the modern diet containing abundant processed food is also an important source of excessive AGEs, chronic exposure to a high-AGEs diet has been used in recent studies, and obvious cardiotoxicities were observed. To avoid the influence of the appetite and other factors on the intake dose of dietary AGEs, we prepared an AGEs solution and administered it quantitatively by the intragastric route to mimic the daily intake of a high-AGEs diet.

Our research showed that chronic ingestion of AGEs solution for 2 months significantly induced the expression of CYP4A and oxidative stress in mouse hearts and in H9c2 cells, as shown by increased cardiac LDH, H_2_O_2_, MDA, and ROS production. Further examination showed that AGEs activated the oxidative pathway with increased expression of NOX2, the main source of ROS in the myocardium, and decreased the activity of SOD, a primary ROS scavenging enzyme. Our data in mice heart are consistent with those of a previous report showing that dietary AGEs are able to catalyze the formation of ROS and H_2_O_2_, leading to the accumulation of oxidative damage in cardiac microvascular endothelial cells and diabetic complications (Giacco and Brownlee, 2010; Liu et al., 2011; Zhang et al., 2018; Wang et al., 2019).

Apoptosis was next examined since AGEs also trigger the cleavage of caspase-3, the pro-apoptotic protein that acts as an indicator of apoptosis, and consequently induce apoptosis in osteoblasts and fibroblasts *via* JNK pathways (Li et al., 2012; Weinberg et al., 2014). In the present study, the AGEs group exhibited significant apoptosis as shown by the TUNEL method. Further examination showed that the expression of phosphorylation at Ser63 and Ser73 of JNK and the expression of c-caspase-3 were all upregulated. Meanwhile, the phosphorylation of Akt and the expression of the anti-apoptotic protein, Bcl-xL, were substantiallly reduced in the AGEs group *in vivo* and *in vitro*. As mentioned above, chronic ingestion of AGEs solution significantly activated the myocardial OS/Apop pathway.

Whether CYP4A is involved in the AGEs-induced OS/Apop pathway was further investigated. Overexpression of CYP4A in HUVECs caused increased levels of intercellular OS (Kang et al., 2006). CYP4A inhibitor reduced ROS generation, NADPH oxidase activity, and NOX protein expression, leading to the amelioration of podocyte apoptosis *in vitro* and *in vivo* in patients with diabetes. Inhibition of CYP4A with an inhibitor or genetic manipulation caused significant reductions in ROS levels in arteries (Singh et al., 2007; Lukaszewicz et al., 2013). In the present study, CYP4A expression in the myocardium of AGEs-treated mice and AGEs-treated H9c2 cells was remarkably elevated concomitant with activation of the OS/Apop pathway. Specific inhibition of CYP4A by HET0016 significantly improved the level of OS/Apop as well as those above mentioned critical factors in the OS/Apop pathway in both AGEs-treated mice and AGEs-treated H9c2 cells, suggesting that CYP4A may mediate the AGEs-induced OS/Apop activation. To confirm the role of CYP4A in AGEs-induced OS/Apop injury, further investigation was performed with the depletion of CYP4A using siRNAs against CYP4a1, CYP4a2, and CYP4a3, since these three types of CYP4a isoforms are all expressed in rat cardiomyocytes (Alsaad et al., 2013). Combined depletion of CYP4A using siRNAs effectively reduced the expression of CYP4A concomitant with the suppression of OS/Apop pathways that were induced by AGEs treatment.

## Conclusions

Chronic intake of excessive AGEs leads to myocardial oxidative stress and apoptosis in healthy mice. CYP4A, up-regulated by AGEs, may mediate the AGEs-activated myocardial OS/Apop pathway. Specific inhibition of CYP4A might be a potential therapeutic option, aiming to prevent and treat chronic myocardial toxicity in persons with diabetes or those consuming a high-AGEs diet.

Further studies may focus on screening drugs with stronger inhibitory effects on CYP4A and investigating the therapeutic effects on AGEs-induced injury. The regulatory mechanism of CYP4A in AGEs-induced myocardial toxicity needed to be clarified. The interaction among AGEs, CYP4A, and peroxisome proliferator-activated receptor γ (PPARγ) will be investigated, since AGEs significantly down-regulated PPARγ expression, and activating PPARγ by atorvastatin or pioglitazone blocked AGEs-induced changes (Yang et al., 2010; Chen et al., 2016).

## Ethics Statement

This study was carried out in accordance with the recommendations of “Animal Experimental Ethics Committee of Dalian Medical University.” The protocol was approved by “Animal Experimental Ethics Committee of Dalian Medical University.”

## Author Contributions

HL, RW, and LW designed the research; RW, LW, JH, SL, XY, PS, YY, JP, JY, and JD conducted the research; RW, LW, SL, and XY analyzed the data; RW and LW wrote the paper, HL revised the paper.

## Funding

This work was supported by the LiaoNing Revitalization Talents Program(XLYC1808031) and the Natural Science Foundation of LiaoNing Province (2015020299).

## Conflict of Interest Statement

The authors declare that the research was conducted in the absence of any commercial or financial relationships that could be construed as a potential conflict of interest.
