# Supplementary material for: Specific Inhibition of CYP4A Alleviates Myocardial Oxidative Stress and Apoptosis Induced by Advanced Glycation End-Products
Source: Front Pharmacol. 2019 Aug 9;10:876. doi: 10.3389/fphar.2019.00876 (PMC6696796; doi:10.3389/fphar.2019.00876)
Supplement: Supplementary file 1 [file Table_1.docx]

Supplemental Data

1. Supplemental methods

1.1 Echocardiography

Transthoracic echocardiography was performed by Vevo 770 system VisualSonics (Toronto, Ontario, Canada) as previously described (Zhang et al., 2018; Chen et al., 2019). At the end of our experiment, animals were anesthetized with isoflurane (3%) and then underwent M-mode echocardiography from a 30 MHz probe. Left ventricle (LV) internal diameter in systole (LVID; s) and diastole (LVID; d), LV posterior wall thickness in systole (LVPW; s) and diastole (LVPW; d) were determined by M-mode echocardiography. Fractional shortening (%FS), ejection fraction (%EF), and LV mass were calculated automatically by an ultrasound machine.

2. Supplemental results

2.1 Inhibition of CYP4A with HET0016 reduced cardiac dysfunction induced by AGEs in mice

AGEs significantly decreased fractional shortening (%FS) and HET0016 treatment significantly improved %FS compared to AGEs group. While AGEs showed tendency to decrease ejection fraction (%EF) and HET0016 increased %EF slightly, but there is no statistical significance. Our results showed that the AGEs induced mild cardiac dysfunction, which was ameliorated by specific inhibition of CYP4A.

|  | Con | Con+HET | AGEs | AGEs+HET |
| --- | --- | --- | --- | --- |
| LVID; d (mm) | 3.75± 0.05 | 3.70± 0.08 | 3.57± 0.06 | 3.61± 0.09 |
| LVID; s (mm) | 1.90 ± 0.07 | 1.88 ± 0.06 | 2.05± 0.12 | 1.98 ± 0.08 |
| LVPW; d (mm) | 0.77 ± 0.03 | 0.78 ± 0.02 | 0.92 ± 0.03^+^ | 0.83 ± 0.03 |
| LVPW; s (mm) | 1.39 ± 0.03 | 1.36 ± 0.03 | 1.21 ± 0.04 | 1.25 ± 0.02 |
| %EF (%) | 85.52 ± 2.35 | 86.67 ± 3.01 | 82.74± 2.89 | 84.18 ± 2.49 |
| %FS (%) | 49.33 ± 2.43 | 49.18 ± 3.12 | 42.57 ± 3.85^+^ | 45.15 ± 2.97^*^ |
| LV mass (mg) | 89.05 ± 1.53 | 87.31 ± 2.09 | 92.63 ± 2.48 | 91.21 ± 2.45 |

Table 3. Inhibition of CYP4A with HET0016 reduced cardiac dysfunction induced by AGEs in mice. Values are means ±S.E.M. (n=9), compared with the Con group, +*p*<0.05; compared with the AGEs group, **p*<0.05 (1-way ANOVA with turkey post hoc).

References

Chen, C., Zou, L.X., Lin, Q.Y., Yan, X., Bi, H.L., Xie, X., et al. (2019). Resveratrol as a new inhibitor of immunoproteasome prevents PTEN degradation and attenuates cardiac hypertrophy after pressure overload. *Redox Biol* 20**,** 390-401. doi: 10.1016/j.redox.2018.10.021.

Zhang, B., Zhang, J., Zhang, C., Zhang, X., Ye, J., Kuang, S., et al. (2018). Notoginsenoside R1 Protects Against Diabetic Cardiomyopathy Through Activating Estrogen Receptor alpha and Its Downstream Signaling. *Front Pharmacol* 9**,** 1227. doi: 10.3389/fphar.2018.01227.
